# Unicornuate uterus with a rudimentary horn diagnosed at scheduled third Cesarean Section

**DOI:** 10.12669/pjms.333.12409

**Published:** 2017

**Authors:** Serkan Bodur, Ulas Fidan, Mehmet Ferdi Kinci, Kazim Emre Karasahin

**Affiliations:** 1 Serkan Bodur, MD. Gulhane Training and Research Hospital, Department of Obstetrics and Gynecology, Ankara, Turkey; 2 Ulas Fidan, Assistant Professor, Gulhane Training and Research Hospital, Department of Obstetrics and Gynecology, Ankara, Turkey; 3 Mehmet Ferdi Kinci, MD. Gulhane Training and Research Hospital, Department of Obstetrics and Gynecology, Ankara, Turkey; 4 Kazim Emre Karasahin, Associate Professor, Gulhane Training and Research Hospital, Department of Obstetrics and Gynecology, Ankara, Turkey

**Keywords:** Rudimentary horn, Unicornuate uterus, Uterine anomaly

## Abstract

A unicornuate uterus with a rudimentary horn is an anomaly caused by defective fusion of the Müllerian duct, estimated to occur in one in 76,000 pregnancies. Life threateningly heavy bleeding is a highly expected clinical consequence of such pregnancies. According to the known literature, only two living twins and few living singleton pregnancies have been reported up to now. Here we report on an incidentally diagnosed unicornuate uterus with a communicating rudimentary horn, found during a cesarean section of a gravida 3, parity 2 (G3 P2) patient. This case is rather unique since the patient has had three full term pregnancies and three cesarean sections without significant fetal compromise. This delivery and the existing literature showed us that extensive uterine correction surgeries need not be automatically proposed when a unicornuate uterus is diagnosed in the preconception period. Such deliveries indicate that women with this uterine anomaly may have the potential to carry pregnancies to full term.

## INTRODUCTION

A unicornuate uterus with a rudimentary horn is an anomaly caused by defective fusion of one of the paired Müllerian ducts. The incidence of rudimentary–horn pregnancy is estimated to be one in 76,000 pregnancies.^1^ Life–threateningly heavy bleeding and abortion are the anticipated outcomes of rudimentary horn pregnancies.^2^,^3^ According to the literature, the newborn survival rate is between 0% and 13% in pregnancies in the rudimentary horn,^4^ with only one third of such gestations reaching term or beyond. Slightly more than 50% of the pregnancies ended with a rupture of the pregnant uterine horn. Although there is no identifiable trend regarding the ruptures, approximately 80% are expected to occur before the third trimester. The maternal mortality rate is volatile—between 6% and 23%.^4^ We were able to find only two live twin and very few live singleton births reported in the literature.^5^ A thorough review of the successful pregnancies showed that few were diagnosed incidentally at delivery rather than being discovered at the preconception period or during routine obstetric care. Here we present an incidentally diagnosed unicornuate uterus with a communicating rudimentary horn. Because of her history of two previous cesarean sections, this patient underwent cesarean section to deliver her third baby at 38 weeks and three days. To our knowledge, there are no prior reports of three viable rudimentary–horn births by one mother without fetal or maternal compromise.

## CASE REPORT

**Fig.1:**
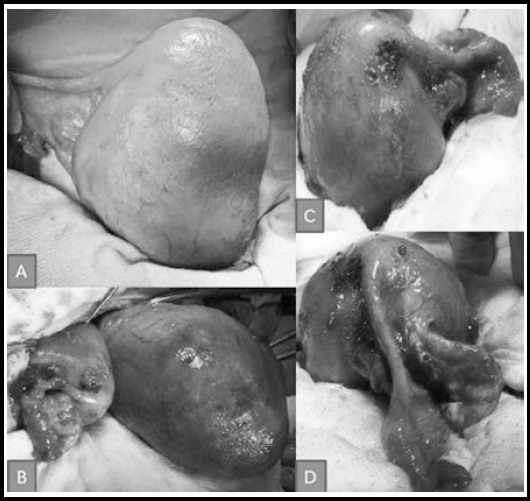
Intraoperative images of the uterus. A cylindrical uterus with a flattened left side wall and absent tubal ostium, left tube and absent left ovary. A rudimentary horn was detected fused with the main cavity from the left-posterior side of the unicornuate uterus together with an attached left tube and left ovary. Rudimentary horn of the uterus. Left tube and ovary attached to the rudimentary horn.

A 33–year–old G3 P2 was seen at the outpatient clinic at 27 weeks of gestation. Her recent pregnancy follow–ups had all been uneventful, according to the medical records. She reported two cesarean sections; the first was performed because of dysfunctional labor, and the second was due to the patient having undergone a previous cesarean section. All three pregnancies were spontaneous. The patient was scheduled for her third cesarean section during an uneventful third trimester, a time when she sought regular prenatal follow–ups. The ultrasonography examination prior to the cesarean section revealed a live fetus with breech presentation and an expected birth weight of 3,300 gr. The Pfannenstiel’s incision was performed, and a baby girl weighing 3,100 gr (Apgar score = 8) was delivered at breech presentation, under spinal anesthesia. On inspection, the uterus was seen to be cylindrical in shape, with a flattened left–side wall and no left tubal ostium, left tube, or left ovary, as can be seen in Fig.1. A rudimentary horn was detected; it was fused to the main cavity on the left–posterior side of the unicornuate uterus. With it was an attached normal right tube and right ovary. The cesarean section was uneventful, and the patient was discharged after the routine 48–hour postoperative clinical follow–up period.

## DISCUSSION

The American Fertility Society designates a unicornuate uterus, which is usually accompanied by a non–communicating rudimentary horn, as a class II uterine anomaly.^4^ Non–communicating rudimentary–horn pregnancies resulting from the migration of sperm to the peritoneum from the unicornuate side of uterus have been reported. Although pregnancies were thought to be more likely to occur on communicating uterine rudimentary horns, Nahum showed that 83% of these pregnancies were located in non–communicating horns, according to a review of 588 cases in the literature.^4^ Our patient was somewhat lucky to have had three pregnancies on the dominating uterine cavity, thereby avoiding the potential hazards associated with pregnancies on the non–dominating cavity. In pregnancies on the non–dominated side, life–threatening hemorrhage often occurs in the second and third trimesters of pregnancy. The worldwide rudimentary–horn–pregnancy experience is uterine rupture in 90% of pregnancies during the second trimester.^4^

Important factors supporting our patient’s full–term pregnancy without significant fetal compromise were the patient’s sufficient blood supply to the dominant uterine cavity and the existence of a possibly normal endometrium within the dominant uterine cavity. Pregnancies located on the rudimentary horn are associated with poor pregnancy outcomes, such as increased rate of missed abortion, intrauterine fetal death and asymmetrical or restricted intrauterine growth due to a decreased blood supply and defective endometrium. The ultrasound–measured distance of the myometrium between the two horns, as well as the variation in thickness of myometrial components of the two horns, both of which facilitate the diagnosis of this anomaly with reasonable accuracy.

The most important reason for the absence of a pre-operative diagnosis was the patient’s lack of prenatal care; her first antenatal visit was at 27 weeks. The suggested diagnostic algorithms were all based on gynecologic ultrasound examinations and magnetic resonance imaging (MRI) findings rather than through routine third-trimester obstetric care. We believed that her relaxed attitude toward obstetric and gynecologic care prevented her from learning of the uterine-anomaly diagnosis during early pregnancy. It must be noted, however, that our patient is not alone with this attitude; we read reports of twin pregnancies—one on each horn—that were incidentally diagnosed during a cesarean section.^5^

If we had not noticed the presence of a cylindrical uterus that was flattened on the left side, along with the absence of the left tube and ovary, we might have missed the presence of the rudimentary uterine horn communicating with uterus at the isthmic level from the left–posterior side, as did the surgeons performing her prior cesarean sections. Honestly, we did not have a knowledge why the previous surgeons had missed the diagnosis but we were assuming that there would be a lack of communication between the surgical team, clinical staff and patient in terms of giving appropriate postoperative information.

We offered to excise the rudimentary horns after the delivery, but there were also reports reporting unfavorable surgical outcomes related with excision of the rudimentary horn like necessity of multiple transfusions, wound dehiscence, and sepsis. Our case is rather unique because our patient had sustained three full–term pregnancies and cesarean sections without significant fetal or maternal compromise, and each of the surgical experiences was uneventful and free from surgical complications.

### Author’s Contributions

***Serkan Bodur*** wasmainly responsible from conception and design of the study. He was also the responsible surgeon of the case.

***Ulas Fidan*** conception and design of the study, literature review.

***Mehmet Ferdi Kinci*** Member of the surgical team, revision of the manuscript.

***Kazim Emre Karasahin*** preparation of the manuscript.
